# Creating healthy workplaces in healthcare: Are we delaying progress by focusing on what we can do rather than what we should do?

**DOI:** 10.3389/fpubh.2023.1105009

**Published:** 2023-03-01

**Authors:** Anthony Montgomery, Olga Lainidi

**Affiliations:** ^1^Department of Psychology, Northumbria University, Newcastle upon Tyne, United Kingdom; ^2^School of Psychology, University of Leeds, Leeds, United Kingdom

**Keywords:** healthy workplaces, mental health of employees, burnout, healthcare, patient safety

## Abstract

All the available evidence points to the fact that healthcare is under considerable stress, and while change is urgently needed there is no quick fix; systemic and sustained changes in organizational cultures within healthcare are required. Moreover, the fragility of healthcare systems globally has been starkly exposed by the Coronavirus 2019 pandemic. We have gathered enough evidence to know what is driving poor wellbeing, and how these processes impact on quality of care and patient safety. Indeed, we have a good idea of what we need to do to improve the situation. Therefore, this begs a simpler question; If we know how to create healthy workplaces, why is it so difficult to achieve this in healthcare? In the following perspective paper, we will argue that we can do better if we address the following three issues: (1) we are ignoring the real problems, (2) limited successes that we are achieving are moving us further from tackling the real problems, (3) culture change is accepted as crucial, but we are not accepting what the evidence is telling us about healthcare culture. Tackling burnout is useful and necessary, but we must increase dignity among healthcare employees. Moreover, we need to train line managers to recognize and facilitate the need of employees to feel competent and be appreciated by others, while helping them set wellbeing boundaries.

## 1. Introduction

All the available evidence points to the fact that healthcare is under considerable stress. For example, in the United Kingdom (UK) a recent report from the International Public Policy Observatory (IPPO) estimates that the financial cost to the NHS of poor wellbeing is £12.1 billion a year (1). Moreover, the fragility of healthcare systems globally has been starkly exposed by the COVID-19 pandemic, with emerging evidence that during COVID healthcare workers from Black, Asian and Minority Ethnic (BAME) groups had a significantly increased risk of mortality when compared to white healthcare workers (2). At a community level, COVID has created a hole in the informal social prescribing networks utilized by primary care to support patients and their families (3). Moreover, the complexity of the COVID problem has been highlighted by recent research indicating that the natural propensity of co-workers to socialize together means that healthcare professionals are at triple the risk of developing COVID-19 infection when comparing co-worker exposures with patient sources (4). Thus, gathering with your work colleagues can be dangerous during a pandemic—even though this maybe the time when you need support most. The pandemic should have alerted policymakers to the fact that creating healthy workplaces is a necessity for the sustainability of healthcare, and an opportunity to address the unavoidable link between healthcare employees' mental health and the quality of healthcare delivery (5).

We have gathered enough evidence in healthcare to know what is driving poor wellbeing, and how these processes impact on quality of care and patient safety (6). Indeed, there is a plethora of studies on the drivers of burnout (7–9), the mechanisms of burnout (10–13) and reviews of burnout interventions in healthcare (14–16). Thus, it could be argued that we have a good idea of what we need to do to improve the situation. However, this begs a simpler question; if there is guidance on how to create healthy workplaces, why is it so difficult to achieve this in healthcare? In the following perspective paper, we will argue that creating healthy workplaces in healthcare can be realized sooner if we address the following three issues: (1) we are ignoring the real problems, (2) limited successes are moving the field further from tackling the real problems, (3) evidence points to the fact that culture change is crucial, but we are not accepting what the evidence is telling us about healthcare culture.

## 2. Are we ignoring the real problems?

Suggesting that researchers are ignoring the real problems is a strong claim. However, a realist review of interventions to promote mental health and happiness among health workers (17) concluded that there are three main barriers. First, lack of engagement from employees was the most common reason for interventions failing; second, the majority of studies were from high-income countries; third, most of the studies targeted one type of healthcare workers (with nurses being the most common). Moreover, a further review of two recent papers—a study protocol (18) and an intervention study (19)—indicates that these problems are continuing. These two recent papers demonstrate an understandable pragmatism toward funding what can be done rather that what should be done. Firstly, the protocol refers to an organizational redesign intended as a system-level intervention, targeting the whole hospital on the level of the organization (18). However, while the intervention is multi-component, it will only target the two major clinical professions—nurses and physicians. The second study (19), which was an intervention study among academic radiologists, demonstrated that self-reported burnout was unchanged or worsened over time across a range of departmental initiatives that were intended to improve culture, workplace efficiency, work-life balance, and personal wellness. The authors reflected on the reasons why the initiatives failed and reported that “our leadership lunches and social events with leadership were poorly attended” (19). These two recent papers mirror the problems identified by the review (17), in showing a focus on a narrow set of employees and a failure to explore the lack of engagement.

The realist review of interventions (17) indicates that interventions have reduced the hospital to the product of its frontline care staff (i.e., physicians and nurses), and ignored the equally important distal roles that impact both the wellbeing of staff and patient safety of a hospital. For example, the out-sourcing of cleaning staff in hospitals, meaning employees are more detached from the organization, has been linked to higher levels of health care–associated infections in both the UK (20) and United States of America (21), with the UK study also indicating that outsourcing was associated with fewer cleaning staff per hospital bed, worse patient perceptions of cleanliness and worse staff perceptions of availability of handwashing facilities. Put simply, it is perverse that organizational interventions place such little importance on the diverse roles that contribute to the effective functioning of a hospital, such as cleaning staff, catering staff, administration staff, healthcare assistants and support staff. Additionally, interventions don't account for the social, economic and historical factors that influence the degree to which staff and patients experience the hospital as an unhealthy environment. Most recently, the obsession with focusing on “what can be done” has been identified as the self-limiting factor in quality improvement initiatives, where improving organizational-level quality measures is prioritized over the healthcare professionals' emotional experience (22). The majority of interventions and protocols reported in high-ranking journals reflect well-designed, pragmatic, doable and measurable approaches to designing interventions. However, the interesting question is whether this pragmatic approach has fallen foul of the social sciences' metaphysical obsession with method—leading to measure-ability always trumping meaningfulness [see Robinson (23), for a full discussion].

To make progress in creating healthy workplaces, the crucial factor is to understand better the most effective way to embed interventions that are informed by bottom-up experiences (not just top-down). Recent evidence from industry indicates that domain experts exhibit a feasibility preference, focusing on the feasibility of a solution as the primary indicator of its quality, while discounting riskier but more novel solutions (24). Thus, in healthcare focusing on what experts perceive as “feasible” rather than what is needed runs the risk of treating the symptoms rather than the drivers of unhealthy workplaces. Congruently, Clarkson (25) describes the typical Plan-do-Study-Act approaches (26) in quality improvement as dangerous on the basis that our approach to healthcare redesign tends to ignore the first element of the design process (i.e., Need, Problem, Solution)—why do we *need* to change something? Accordingly, healthcare can rush toward problems and solutions, which is probably rooted in the pathogenic approach to training. This leapfrogging over the “need” is characteristic of our approach to burnout in healthcare.

**Figure 1:**
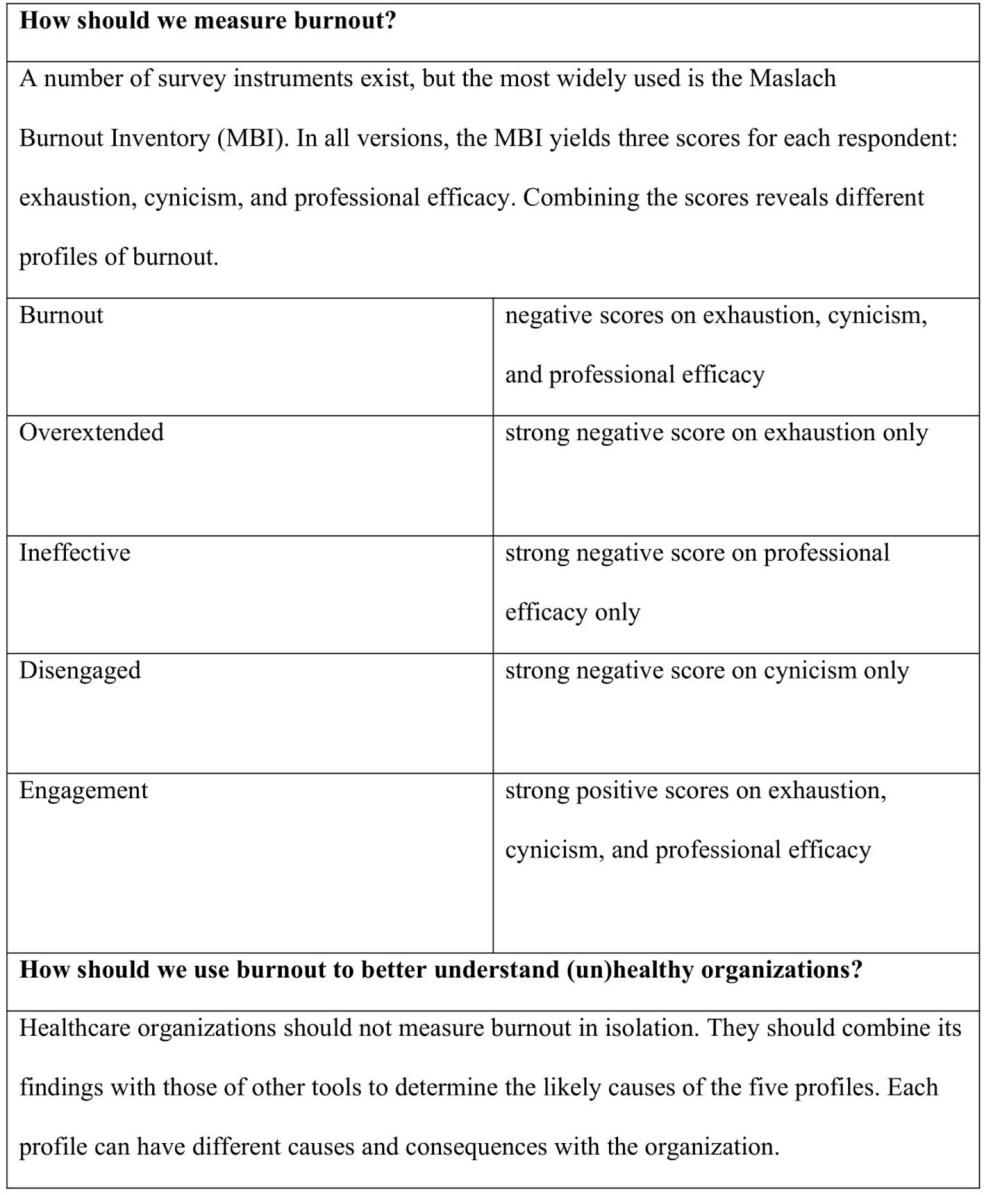
Profiles of burnout (27).

Burnout should be considered as a symptom of a dysfunctional organizational system, a starting point not an end one, inviting us to investigate why the workplace is unhealthy. Assessing the range of burnout profiles (see Figure 1) rather than a simplistic burnout/no burnout dichotomy is a more sensible and evidence-based approach to burnout. There is a need to move from a burnout-centric approach in healthcare to a healthy workplace-centric one, especially when one considers that only 10–15% of employees fit the true burnout profile (as measured by the Maslach Burnout Inventory), meaning if interventions target heterogenous groups, they are mudding the water as to their efficacy (27). Burnout prevalence rates within healthcare can vary considerably among organizations and medical specialties from 17 to 69%, (1), and this compounds the problem of comparison, as different approaches to classifying burnout have been used in healthcare (28). Assessing the different profiles of burnout (see Figure 1) enables the investigation of factors driving each of the different profiles. For example, developing interventions that seek to address employees' need for fairness will look very different from those seeking to address employees' need for a sense of community in a workplace.

## 3. Are limited successes problematic?

Interventions that result in limited success (e.g., reduced burnout among front-line staff) risk becoming the victim of their own success—meaning that healthcare organizations will be less motivated to engage in the long-term sustainable goal of healthy workplaces if we can extinguish some fires at the front-line. There is a reality outside of well-designed interventions where healthcare workers need to use foodbanks and will be less able to meet rising energy bills (especially in the UK) (29). Income inequality is an environmental stressor that is associated with higher levels of burnout, with research indicating that psychological demands placed on employees as a result of job insecurity are compounded when they occur in a context of economic inequality (30). Equally, financial stress has been linked with suicidal thoughts and behaviors in the general working population (31). Indeed, organization-level interventions (aiming to increase employee control) do not protect employees from poor working conditions (32). Congruently, data from the first wave of the pandemic among UK health workers indicated that groups at higher risk of experiencing poor mental health included women, younger staff and nurses (33), meaning those most likely to have less resources and greater demands. Should the approaches to healthcare wellbeing not also address the precarity pandemic that is a significant source of stress for a large number of healthcare employees? What is the point of burnout interventions if the main cause of stress is day-to-day survival in and out of work?

While the full fallout of COVID-19 is not yet known, there is some initial evidence that people have recalibrated their needs, and that healthcare workers feel that they are quite literally on the front-line, alone (34). The recent evidence on the impact of COVID indicates increased anxiety and stress among both clinicians and affiliated health workers (35–37) which can't augur well for patients in the future. The pandemic has revealed that policymakers and healthcare organizations need to become much better at protecting the wellbeing of healthcare workers, regardless of the cost. Evidence that on some occasions the needed Personal Protective Equipment (PPE) couldn't be provided during the pandemic, and staff were requested to be silent about it (38, 39), must have sent a powerful signal about the expectations that healthcare staff would keep going regardless of the pandemic. Moreover, the macroeconomic measures necessitated by the pandemic (e.g., Furlough schemes) indicated that money can be found when it is really needed. These aforementioned aspects of the pandemic make it easier to appreciate why healthcare workers are considering a career change and/or weighing up their commitment to their job (40–42).

Local change initiatives in healthcare organizations focused on a narrow range of day-to-day working practices will not provide the impetus needed to signal to healthcare workers that wellbeing and dignity are valued priorities. A review of the evidence on how changes to organizational and management practices can improve staff wellbeing in healthcare and primary care reveals a dearth of whole organization approaches and lack of long-term evaluation (1). Simply repeating the obvious fact that better designed interventions are needed (i.e., longitudinal studies) masks a more interesting inquiry into why healthcare staff would be reluctant to participate in wellbeing initiatives in the first place and don't have the energy, time and inclination to be “dragged” into more extensive full-scale efforts (43, 44). In this regard, the willingness of individuals to be a facilitator in an intervention is highlighted by a recently published cluster randomized control trial of mindfulness to reduce mental health problems and promote wellbeing in UK schools (45). No evidence was found to support the use of mindfulness with school children, but the interesting aspect of the research was the reflection of the researchers that teachers may not have been the best people to deliver the intervention (for a myriad of reasons). The trial, which was methodologically rigorous, is a good example of how ignoring the context can condemn a promising intervention. This chimes with a recent realistic review of interventions to improve wellbeing and decrease burn-out among critical care healthcare professionals (46), which found that contextual factors such as ethnicity, workload, and work schedules played a crucial role in determining the effectiveness of interventions. More specifically, the detailed review highlighted the need to tailor interventions according to the reported needs of participants and incorporate authentic engagement. As the old adage goes, no plan survives contact with the enemy – the reality of healthcare can unmask the limitations even of the most rigorously designed intervention.

## 4. Culture change is crucial, but we are not accepting what the evidence is telling us about healthcare culture

Healthcare workers are highly intrinsically motivated to do their job and positively impact the patients they serve (47–49). However, healthcare workers are especially prone to the detrimental effects of emotionally disturbing work, due to the higher call intensity of their occupation (50). The drive for healthcare employees to “keep going” and “get the job done” has a dark side referred to as pathological altruism, which includes behaviors that attempt to promote the welfare of another but can have pernicious long-term consequences for the care giver (51). Healthcare can exploit the professional ethic of healthcare professionals which results in a form of dysfunctional professionalism that supports maladaptive healthcare structures in education and practice (52), and which can influence staff at all levels. Is it any surprise that these highly motivated individuals feel numb toward interventions that seek to increase their “resilience” or “engagement”?

Employee silence in healthcare is a big elephant in the room (53). Healthcare is not yet ready to deal with a basic conundrum about organizational behavior; why do professionals who are highly dedicated to their work choose to remain silent on critical issues that they recognize as being professionally and organizationally significant? Speaking up interventions in healthcare achieve disappointing outcomes because of a professional and organizational culture which is not supportive of speaking-up (54). Healthcare employees understand that Healthcare is intolerant of mistakes, and leaders are rewarded for “moving on” before the extent of the problem becomes un-manageable (55). Thus, interventions and policies, which do not address or account for the range of economic and social conditions pushing the need to “move on” are unlikely to succeed, especially among a healthcare workforce that feels undervalued, underpaid and under-supported. Additionally, few studies have fully exploited the potential of Public-Patient Involvement (PPI) and the co-production of interventions and policies with the relevant stakeholders [see Taylor et al. (56), for a good example of PPI involvement]. Co-production with interdisciplinary stakeholders has the potential to create a different narrative concerning healthcare worker wellbeing where healthcare delivery is connected with staff wellbeing, and patients can be at the forefront of demanding that healthcare workers are not required to “keep going”—no matter what.

## 5. What can we do differently?

The three questions tackled in this perspective paper—ignoring whole organization solutions, limited successes that address symptoms not causes, and an inability to deal with deeply embedded cultural problems (e.g., employee silence, the hidden curriculum) —are inhibiting progress toward creating healthy workplaces. Burnout is a clue that we are not valuing or adequately protecting the wellbeing of a highly motivated workforce. Tackling burnout is useful and necessary, but the bigger challenge is to increase dignity among healthcare employees, no matter how difficult it is to measure and translate it into a policy and/or an intervention. A good starting point is to utilize the fact that healthcare leaders at all levels are *de facto* acting as “sensegivers” and “sensemakers” for their employees (53). Congruently, there is a need to train line managers how to set wellbeing boundaries (e.g., taking adequate breaks) for themselves and their employees. Not even a pandemic has transformed the level of resources that healthcare workers and patients deserve, beyond symbolic clapping (57).

The problems that have been identified in the Quality Improvement (QI) field in healthcare are relevant to creating healthy workplaces. For example, QI has made notable progress in many areas, but the holes in QI have been identified as: too much attention to individual professional behavior over systems, less attention of QI in mental illness and disability, the ongoing muting regarding social inequalities in healthcare and the lack of research and evaluation in education and training (58). Not repeating the mistakes of QI when creating healthy workplaces can inform our approach. Progress in creating healthy workplaces can be achieved if we accept the advice of Greenhalgh (59) that we should never abandon the narrative-interpretive paradigm and try to get by on “evidence” alone and we should engage pragmatically with the multiple uncertainties involved and offer a flexible and emergent approach to exploring them (60).

Creating healthy work environments within healthcare needs to appreciate the unique way that healthcare professionals are educated and socialized (61). For example, a common experience among healthcare staff is a feeling that they are unable to share their concerns, and their managers are reluctant to have honest informal conversations, a situation exacerbated by social distancing required during the pandemic. Thus, silence is the result of such “protective hesitancy” as both may not feel it is “psychologically safe” to have such discussions (44, 62, 63). The hidden curriculum suggests that the induction period for many young physicians is characterized by a toxic performance culture, whereby adversity is viewed as “character building” and emotional repression is valorized (64, 65), that results in medical students reporting inaction in the face of emotionally challenging situations (66, 67). Regulatory bodies have an important role to play here, and in this sense, can be virtual components of the organization; thus, we need them as full partners in creating healthy workplaces. Only “whole organization” approaches that include all staff and stakeholders have the potential to sustainably address the worker wellbeing public health challenge.

We can do two things differently going forward. Firstly, the three problems we have identified reflect the fact that healthcare can suffer from a silo-mentality. There is a significant lack of experience in genuine interdisciplinary collaboration by the relevant scientific fields (i.e., public health, architecture, occupational health, ergonomics, nutrition, etc.,) on building healthy workplaces. This is a good place to start. Secondly, individual approaches to the problem have dominated in comparison to organizational approaches, with the latter considered complex and difficult to implement. Let's make them less complex by co-producing them with healthcare employees and patients to see if they are actually feasible.

## Data availability statement

The original contributions presented in the study are included in the article/supplementary material, further inquiries can be directed to the corresponding author.

## Author contributions

AM and OL contributed to the conception and writing of this paper. Both authors contributed to the article and approved the submitted version.
